# The Use of eHealth and Provider-Based Health Services by Patients with Diabetes Mellitus: Protocol for a Cross-Sectional Study

**DOI:** 10.2196/resprot.6529

**Published:** 2016-10-31

**Authors:** Anne Helen Hansen, Meghan Bradway, Jan Broz, Tor Claudi, Øystein Henriksen, Silje C Wangberg, Eirik Årsand

**Affiliations:** ^1^ University Hospital of North Norway Tromsø Norway; ^2^ Department of Community Medicine Faculty of Health Sciences UiT - The Arctic University of Norway Tromsø Norway; ^3^ Norwegian Centre for E-health Research University Hospital of North Norway Tromsø Norway; ^4^ Department of Internal Medicine Second Faculty of Medicine Charles University Prague Czech Republic; ^5^ Medical Centre Nordland Hospital Bodø Norway; ^6^ Faculty of Social Sciences Nord University Bodø Norway; ^7^ Department of Substance Use and Mental Health University Hospital of North Norway Narvik Norway; ^8^ Department of Health and Care Sciences Faculty of Health Sciences UiT - The Arctic University of Norway Narvik Norway; ^9^ Department of Clinical Medicine UiT - The Arctic University of Norway Tromsø Norway

**Keywords:** use of eHealth, health care utilization, cross-sectional study, diabetes mellitus, socioeconomic status, Norway

## Abstract

**Background:**

The prevalence of diabetes and the use of electronic health (eHealth) resources are increasing. People with diabetes need frequent monitoring and follow-up of health parameters, and eHealth services can be of great significance in this regard. However, little is known about the extent to which different kinds of eHealth tools are used, and how the use of eHealth is associated with the use of provider-based health care services among people with diabetes.

**Objective:**

The primary objective of this study is to investigate the use of eHealth and its association with the use of provider-based health care services. The secondary objectives include investigating which eHealth services are used (apps, search engines, video services, social media), the relationship between socioeconomic status and the use of different eHealth tools, whether the use of eHealth is discussed in the clinical encounter, and whether such tools might lead to (or prevent) doctor visits and referrals.

**Methods:**

We will conduct cross-sectional studies based on self-reported questionnaire data from the population-based seventh Tromsø Study. Participants will be diabetic patients aged 40 years and older. According to our estimates, approximately 1050 participants will be eligible for inclusion. Data will be analyzed using descriptive statistics, chi-square tests, and univariable and multivariable logistic regressions.

**Results:**

The grant proposal for this study was approved by the Northern Norway Regional Health Authority on November 23, 2015 (HST 1306-16). Recruitment of participants for the Tromsø Study started in 2015 and will continue throughout 2016. This particular project started on July 1, 2016.

**Conclusions:**

This project may yield benefits for patients, health care providers, hospitals, and society as a whole. Benefits are related to improved prevention services, health, experience of care services, self-management tools and services, organizational structures, efficiency of specialist care use, allocation of resources, and understanding of how to meet the challenges from the increasing prevalence of diabetes. This project has potential for generalization to other groups with chronic disease.

## Introduction

Solutions based on information and communication technology for health information, self-management, and novel treatment strategies have developed rapidly in recent years. These solutions have not only become an option for patient self-management, but also potential aids to health care services in their struggle to keep up with the population’s increasing expectations of service. Of particular interest are patients with chronic diseases, such as diabetes mellitus (DM), who are in need of frequent monitoring and follow-up of health parameters.

### Increasing Prevalence of Diabetes Mellitus

The prevalence of diabetes is increasing. In 2014 approximately 422 million adults worldwide were living with diabetes [1]. The global prevalence of DM is estimated at 8.5% [1], and 4.3% in Norway [2]. However, since many cases are undiagnosed, it is difficult to estimate the precise prevalence. The World Health Organization has declared diabetes one of the *big five* chronic diseases [3]. Patients represent a large proportion of health care contacts, and costs attributable to DM represent approximately 1.4% of total Norwegian expenditure on health care [4]. Diabetes is a considerable burden on patients in terms of morbidity and mortality, and only one out of eight patients reach the combined national treatment targets for prevention of diabetic complications [5]. Norway wants to become a leading country in the prevention, treatment, and follow-up of diabetes [6]. Type 1 diabetes mellitus (T1DM) and type 2 diabetes mellitus (T2DM) account for approximately 20% and 80% of cases, respectively.

### Increasing Use of eHealth Services

Electronic health (eHealth) refers to “the transfer of health resources and health care by electronic means” [7], and the Internet plays a major role in eHealth interventions. In the joint population of seven European counties, 44% of the total population reported using the Internet for health purposes in 2005 [8,9], increasing to 52.2% by 2007 [10]. In Poland, which is consistent with European trends, 66.7% of the population reported using the Internet for health purposes in 2012 [11]. Among Internet users in the United States and Europe, approximately three quarters conduct health-related searches [8-10,12]. Most Norwegian households (97%) had Internet access in 2015 [13], and 78% of the population 15 years and older have reported using the Internet for health purposes [14].

### Relationships Between the Use of eHealth and the Use of Provider-Based Health Services

The use of eHealth is reported to be positively associated with general practitioner (GP) visits (yes/no) [9], but not associated or inversely associated with the frequency of regular provider visits [15,16]. Patients might use eHealth before the visit to seek information and/or to decide about the need to see a doctor, and after the visit for reassurance or additional information [17]. It has been stated that the use of eHealth may postpone or replace medical consultations [18], and approximately 30% of eHealth users in France (aged 15-30 years) reported that they often used the Internet instead of visiting a doctor [19]. In the same study, 88.6% of respondents reported that eHealth use did not change their consultation frequency, whereas 4.9% reported seeing a doctor more often, and 6.5% less often. This trend might differ in older populations, since eHealth use is inversely associated with age [9,10,14,20,21]. A German study found that heavy users of health services were 73% more likely to seek health information on the Internet compared to nonusers [12]. This finding conforms with the illness behavior model [22], indicating that people in poor health are more likely to seek disease-related information online and use health care services to a larger extent. Whether eHealth use in a Norwegian population with diabetes might be associated with the use of provider-based health care services has yet to be explored. Theoretically, eHealth use might enhance self-management and reduce health care visits and costs, based upon the assumption that prevention and treatment of diabetic complications (which arise due to poor disease control) represent a large burden of disease and substantial costs for health care services and society [23-25].

The use of eHealth services often takes place without doctor involvement. A study from the United States found that only 31% of users of mobile health (mHealth; mobile and wireless communication technologies that aid in health and health care) prioritized their physician’s involvement [20]. The extent to which DM patients in Norway discuss the information they find on the Internet with their doctors is unknown.

### Socioeconomic Status and the Use of eHealth

Health care services and communication inequalities may contribute to inequalities in health, as it is well known that new interventions and treatments reach people in higher socioeconomic groups first [26,27]. Research indicates that women, younger people, and people with middle and high socioeconomic status (SES) are more likely to seek health information and advice from the Internet [9,22,28,29]. Andreassen et al showed that both long-term illness and good health were positively associated with eHealth use in Europe [9], partly contradicting the illness behavior model [22]. In the case of DM, however, there is evidence that there is no immediate gain from implementing health technology platforms in less advantaged groups, in contrast to middle (and especially higher) educated groups, with possible consequences regarding health outcomes [28]. Regarding self-initiated use, Wangberg et al found that SES is related to differential use of eHealth, as people with higher education use eHealth tools that are more likely to influence health behaviors [30]. To our knowledge, the relationship between SES and what kinds of eHealth tools people with DM use has yet to be studied.

### Planning for Future eHealth and Provider-Based Health Care Services

Teams at the Norwegian Centre for E-health Research have been developing and studying Internet-based self-management tools for chronic diseases (including DM) for more than a decade, with the understanding that communication technologies hold great potential for making health services and diabetes care more effective and efficient. However, this potential has not been fully realized. Research on the combined use of consumer-based eHealth (as defined by the use of apps, search engines, video services, and social media) and provider-based health care, as well as the interaction between the use of these different sources of care, is scarce [24,31].

eHealth is an area of continuous and rapid development, and the use of eHealth services and their possible associations with health care utilization and socioeconomic position are likely to vary between regions, countries, diagnostic groups, health care services, and health care systems. Hence, research from different cultural and economic settings is important to achieve an overall epidemiological view and comprehensive understanding, as well as to identify vulnerable subgroups. Understanding the influence of eHealth on health care utilization in the large and growing group of patients with diabetes is important for patients, health care providers, administrators, policy makers, and society, in order to enable evidence-based planning for future eHealth and provider-based health care services, thereby providing better health outcomes.

## Methods

### Objectives

The primary objective is to investigate the use of eHealth in Tromsø, Norway, and its association with the use of provider-based health care services. This study will peruse the following specific objectives:

1. To investigate which eHealth services are used.

2. To investigate whether the use of eHealth is associated with the use of primary and specialist health care services.

3. To investigate whether the use of eHealth might lead to or prevent doctor visits.

4. To investigate whether patients’ use of eHealth is discussed in clinical encounters.

5. To investigate whether the use of eHealth is associated with referrals to specialist care.

6. To investigate whether the use of eHealth is associated with SES.

7. To assess how knowledge gained from this study can be used to develop and implement eHealth solutions and changes in health care services, in order to increase DM patients’ chances of reaching their treatment goals.

Based on previous research and the authors’ experiences, the following hypotheses have been generated:

1. The use of eHealth is positively associated with one or more GP visits during a year.

2. The use of eHealth is not associated with the use of emergency departments.

3. The use of eHealth does not have any impact on the frequency of doctor visits.

4. It is more common for eHealth users not to discuss the use of eHealth in the consultation than to discuss it.

5. The use of eHealth is associated with an increase in GPs’ referrals to specialist services.

6. The use of eHealth is positively associated with the use of outpatient specialist services.

7. The use of eHealth is inversely associated with hospital admissions.

8. The use of eHealth is positively associated with higher SES.

These hypotheses will be tested using data from the Tromsø Study, which will be analyzed using descriptive statistics, chi-square tests, and logistic regressions.

### Study Population

Population-based health surveys have been conducted in Tromsø, Norway since 1974. We plan to retrieve questionnaire data from the seventh cross-sectional Tromsø Study (Tromsø 7), conducted in 2015-2016. Data will be available at the beginning of 2017. We have participated in the design of the survey, which for the first time includes self-reported data on the use of eHealth. Tromsø 7 also includes data on referrals, the use of primary and specialist health services, self-rated health, diseases and symptoms, use of medication, as well as demographic and socioeconomic data. The following question will be particularly important in our study: *Based on the information you have found on the Internet,have you decided (1) to consult a doctor, or (2) not to consult a doctor?* Participants were also asked if they discussed the information they gained from the Internet with their doctor.

Tromsø is the largest city in Northern Norway with 75,762 inhabitants (as of January 1, 2016). All residents aged 40 years and over (approximately 33,000 persons) were invited to participate in Tromsø 7; more details about Tromsø 7 are available at the Tromsø Study website [32]. Assuming a participation rate of 65%, 21,450 individuals will attend Tromsø 7. In the sixth Tromsø Study (Tromsø 6), conducted in 2007-2008, 4.9% of the participants (30 years and older) reported having diabetes (T1DM or T2DM). We therefore assume that approximately 1050 individuals with diabetes will constitute the study sample. The prevalence of diabetes has increased in recent years, and the Tromsø 7 participants (aged >40 years) are older than the Tromsø 6 participants (aged >30 years), making it likely that the sample will be larger.

### Study Design

The study design will be cross-sectional. Data will be analyzed using descriptive statistics, chi-square tests, and univariable and multivariable logistic regressions. The following variables will be available for adjustment in the multivariable regression models: age, gender, marital status, education, income, employment status, duration of the GP-patient relationship, frequency of GP visits, patients’ assessment regarding doctor visits due to the use of eHealth, if eHealth information has been discussed with the doctor, self-rated health, chronic diseases, and the Euro Quality of Life Group five dimensions scoring scale [33]. Analyses will be performed using the newest version of Stata.

## Results

The grant proposal for this protocol was approved by the Northern Norway Regional Health Authority on November 23, 2015 (HST 1306-16). Tromsø 7 has been approved by the Regional Committee for Medical and Health Research Ethics (REK; 2014/940). Recruitment of participants started in March 2015 and is currently ongoing. Written informed consent was obtained from all participants. Data will be available for analyses at the beginning of 2017. This project has been presented to the REK, which found that an application was not required according to the Norwegian Health Research Act (2015/1779/REK nord). This particular project started on July 1, 2016.

## Discussion

We expect that our eight hypotheses will be confirmed during the conduct of the studies. For patients, the expected benefits of the project will be improved prevention services and prospects for improved health, improved experience of care services, and improved support for self-management tools and services. For health care providers, benefits will include improved organizational structures, tools, and services for diabetes care. Expected benefits for hospitals will be a more efficient use of specialist care, and allocation of resources for other activities. For society, benefits will include better understanding of how to meet the challenges from the increasing prevalence of diabetes, and better utilization of novel health technologies. This project has the potential to be generalized to other groups with chronic diseases.

## Abbreviations

DM: diabetes mellitus
eHealth: electronic health
GP: general practitioner
mHealth: mobile health
REK: Regional Committee for Medical and Health Research Ethics
SES: socioeconomic status
T1DM: type 1 diabetes mellitus
T2DM: type 2 diabetes mellitus
